# A dose-escalation study of HP501, a highly selective URAT1 inhibitor, in male Chinese patients with hyperuricemia

**DOI:** 10.1038/s41598-023-49052-x

**Published:** 2023-12-14

**Authors:** Ruilin Ding, Xuehong Deng, Longxia Chen, Yang Zhen, Xinghai Li, Tengqiong Xiong, Yuanhua Zhang, Hong Chen, Xiaojing Hu, Yun Li, Yi Zhou, Feng Jiang, Qing Peng, Xiaojie Wang

**Affiliations:** 1 Institute of Drug Clinical Trial/GCP Center, The Affiliated Hospital of Southwest Medical University, Luzhou, 646000 Sichuan China; 2 Clinical Pharmacology Research Center, Chengdu Fifth People’s Hospital, Chengdu, 610041 Sichuan China; 3 Hinova Pharmaceuticals Inc., Chengdu, 610041 Sichuan China; 4 Department of Cardiology, The Affiliated Hospital of Southwest Medical University, No. 25 Taiping Street, Jiangyang District, Luzhou, 646000 Sichuan China; 5 Department of Endocrinology, The Affiliated Hospital of Southwest Medical University, No. 25 Taiping Street, Jiangyang District, Luzhou, 646000 Sichuan China

**Keywords:** Drug discovery, Medical research, Molecular medicine

## Abstract

HP501 is a highly selective renal urate transporter 1 (URAT1) inhibitor used for treating hyperuricemia. This study aimed to evaluate the tolerability, pharmacokinetics, and pharmacodynamics of HP501 in male Chinese patients. Patients with hyperuricemia were sequentially assigned to receive oral doses of HP501 (30, 50, 60, 90, and 120 mg) as a single dose on Day 1 and as once-daily doses from Days 4 to 13. Safety, pharmacokinetic, and pharmacodynamic data were collected. Multiple oral doses of HP501 were well-tolerated in all the cohorts. The most common adverse events (≥ 10% of patients) of any grade regardless of drug relationship were gout flare (14 patients, 25.93%), diarrhea (12 patients, 22.22%), elevated ALT (8 patients, 14.81%), hypertriglyceridemia (7 patients, 12.96%), dry mouth (7 patients, 12.96%) and oral ulcer (7 patients, 12.96%). All adverse events were mild or moderate. The C_max_ and exposure (AUC) of HP501 was approximately dose-proportional between 30 and 120 mg. A dose-dependent serum uric acid (UA)-lowering effect was observed in the dose range of 30 to 60 mg and the serum UA lowering effect was similar between 90 and 120 mg on day 13, indicating that the maximal serum UA lowering effect of HP501 was achieved at 90 mg in the patients with hyperuricemia. In conclusion, the tolerability, pharmacokinetics, and pharmacodynamics supported 90 mg HP501 for subsequent clinical studies of this highly selective URAT1 inhibitor.

Clinical Trial registration: No. CTR20212259 (http://www.chinadrugtrials.org.cn/) was registered in September 2021, and No. CTR20222257 was registered in September 2022.

## Introduction

Over 20% of people in high-income countries have been affected by hyperuricemia^1,2^. In China, the total hyperuricemia prevalence was 14% between 2018 and 2019^3^. Hyperuricemia may lead to the deposition of monosodium urate crystals in the joint structures, thereby increasing the risk of gout. Gout is a common inflammatory arthritis affecting 10% of adults^4^. In addition to causing excruciating arthritic pain, gout is related to a subset of comorbidities, including nephropathy, type 2 diabetes, hypertension, and heart disease^5^. It has been reported that gout is associated with a 17% higher all-cause mortality risk than those without gout^6^. For the management of gout, using serum uric acid (UA)-lowering therapy to decrease the overall urate burden is effective and helpful.

Drugs for hyperuricemia treatment include xanthine oxidase inhibitors (XOIs), which block urate production, and uricosuric drugs, which promote the urinary excretion of UA. Allopurinol and febuxostat are the commonly used XOIs. However, the target serum UA level (serum UA ≤ 360 μmol/L) cannot be achieved in over 30% of patients with hyperuricemia who receive XOI alone^7^. Urate transporter 1 (URAT1) is a membrane transporter that mediates most of the re-absorption of UA in kidneys^8^. Uricosuric drugs target URAT1, thereby treating hyperuricemia^9^. Benzbromarone was the first approved URAT1 inhibitor; however, it has been withdrawn from Western countries owing to serious hepatotoxicity^10^. Lesinurad has received FDA approval for the treatment of hyperuricemia; however, it has a high risk of nephrotoxicity and withdrawn in the US in 2019^11^. Another selective URAT1 inhibitor, dotinurad, has been approved in Japan. Therefore, URAT1 inhibitors for treating hyperuricemia are greatly needed worldwide.

HP501, a highly selective URAT1 inhibitor, was developed by Hinova Pharmaceuticals, Inc. (Chengdu, China). During phase I/IIa trial that enrolled 68 healthy volunteers and 36 patients with hyperuricemia, HP501 significantly reduced serum UA levels with low incidence of adverse events (AEs)^12^. In the single ascending dose (SAD) part of the study, exposure to HP501 increased with increasing HP501 dose from 5 to 60 mg in healthy volunteers^12^. In the multiple ascending dose (MAD) part, urinary excretion of serum UA dose-dependently increased in the 3–45 mg dose range^12^. Preclinical data showed that the no-observed-adverse-effect levels of HP501 in rats and monkeys were 30 and 100 mg/kg, respectively (unpublished data). The human equivalent doses (HEDs) could be reached to 200 mg/d. Therefore, the plasma exposure and serum UA-lowering effects of HP501 may not reach saturation at dose levels of 45–60 mg. Thus, we conducted this dose-escalation study to further evaluate the tolerability, pharmacokinetics, and pharmacodynamics of HP501 in male Chinese patients.

## Methods

### Study design and treatment

The Institutional Ethics Committees of the Affiliated Hospital of Southwest Medical University (Luzhou, China; approval number: L2022040) and Chengdu Fifth People’s Hospital (Chengdu, China; approval number: 2021-013-01) approved this study. Written informed consent was obtained from all the patients.

This dose-escalation study included an approximately 14-day screening period, a 2-day run-in period, a 3-day SAD period, and a 13-day MAD period. Seven dose cohorts (30 mg, 50 mg, 60 mg, 90 mg, 120 mg, 150 mg, and 180 mg) were studied. Following the screening period, eligible patients were admitted to the hospital on Day − 2 for diet control (the run-in period). Then, the patients were sequentially assigned to each dose cohort before treatment. During the SAD period, the patients were administered a single dose of HP501 on Day 1 under fasting conditions and observed for 3 days for tolerability. If patients had acceptable safety in the SAD test, the MAD test was conducted. The patients received HP501 once daily from Days 4 to 13 under fasting conditions for 10 days. Dose escalation was performed only after the previous dose was assessed for acceptable safety and tolerability.

### Patients

The inclusion criteria included the following: hyperuricemic patients aged 18–65 years old with or without gout, diagnosed according to the Chinese guideline^13^; serum UA levels ≥ 540 μmol/L for patients with asymptomatic hyperuricemia or ≥ 480 μmol/L for patients with gout.

The main exclusion criteria included the following: patients with a body mass index (BMI) below 18 or over 30 kg/m^2^; patients with a known history of allergy to the preparations of HP501; patients who had participated in the prior clinical trials of HP501; patients who had administrated serum UA-lowering drugs in the 14 days before enrollment; patients who had used any drug or food that can either affect CYP2C8 or CYP2C9 in the 14 days before enrollment; patients with a peptic ulcer; patients with serious cardiac disorders; patients with abnormal laboratory test results, such as an estimated glomerular filtration rate < 60 mL/min/1.73 m^2^, alanine aminotransferase (ALT) or aspartate aminotransferase (AST) ≥ 2 × upper limit of normal (ULN), and hemoglobin < 90 g/L.

### Study endpoints

The primary endpoints of the study were safety and tolerability. The secondary endpoints included pharmacokinetics, efficacy, and pharmacodynamics.

### Safety assessments

Twelve-lead electrocardiography (ECG), vital signs (respiration, blood pressure, pulse, and temperature), physical examinations, and laboratory tests (routine urine tests, routine blood tests, blood biochemical tests, and serum and urinary beta 2-microglobulin) were used to evaluate the safety and tolerability of HP501. All AEs and serious AEs (SAEs), as well as their severities, were recorded. The AEs were coded using the Medical Dictionary for Regulatory Activities (version 24.0).

### Pharmacokinetic evaluation

Blood samples for pharmacokinetic evaluation were collected predose and at 1, 2, 3, 4, 5, 6, 8, 10, 12, 24, 48, and 72 h postdose on Day 1; predose on Days 5–12; and predose and at 1, 2, 3, 4, 5, 6, 8, 10, 12, 24, 48, and 72 h after the final dose on day 13. The collected blood samples (4 mL) were centrifuged at 2000 × *g* at 4 °C for 10 min. Plasma samples were shipped to Chongqing Denali Medpharma Co., Ltd. (Chongqing, China) for analysis.

Plasma concentrations of HP501 were determined by liquid chromatography–tandem mass spectrometry (LC-MS/MS) using an LC-30AD chromatograph (Shimadzu, Kyoto, Japan) and an API 5500 mass spectrometer (AB Sciex, Framingham, MA, United States) with HC-1239 (provided by Hinova Pharmaceuticals Inc.) as the internal standard. The lower limit of quantification was 5 ng/mL for HP501, with a calibration range of up to 5000 ng/mL.

All the pharmacokinetic parameters were determined by non-compartmental analysis (NCA) using Phoenix WinNonlin software (version 8.1; Certara, Princeton, NJ, United States). The key pharmacokinetic parameters included maximum plasma concentration (C_max_), time to C_max_ (T_max_), area under the concentration-time curve from time zero to the last measurable concentration (AUC_0-t_), from time zero to infinity (AUC_0-∞_), terminal elimination phase half-life (t_1/2_), clearance rate (CL/F), apparent volume of distribution at terminal phase (Vz/F), maximal and minimum concentration during one dosing interval τ at steady state (C_max,ss_ and C_min,ss_), area under the concentration-time curve during one dosing interval τ at steady state (AUC_τ_) and accumulation ratio (R_ac_).

### Pharmacodynamic analysis

Blood samples for pharmacodynamic analysis were collected predose and at 2, 4, 8, 12, 24 and 72 h postdose on Day 1; predose on Days 5–12; and predose and at 2, 4, 8, 12, 24, 48, and 72 h postdose on Day 13. All blood samples (2 mL each) were collected at the Affiliated Hospital of Southwest Medical University to measure serum UA levels.

The pharmacodynamic parameters included the maximum percent change in serum UA level from baseline (ΔC_max_%), the area under the mean percent change curve from 0 to 24 h (ΔAUC_0-24 h_) and the time to the maximum percent change in serum UA level from baseline (T_sUA,max_).

### Statistical analysis

SAS software (version 9.4; SAS Institute, Cary, NC, USA) was used for statistical analyses. Demographic, pharmacokinetic, pharmacodynamic, and safety data were summarized as descriptive statistics. The safety analysis was performed on the safety population, which included patients who received at least 1 dose of HP501. χ^2^ test was used to compare the incidences of AEs among the cohorts. The pharmacokinetic analysis was performed in patients who received HP501 and had at least 1 post-dose measurement of plasma HP501. The pharmacodynamic analysis was performed in patients who received HP501 and had at least 1 post-dose measurement of serum UA level. The sample size of this study was determined empirically. Cohorts of 8–12 patients were considered adequate to initially characterize the safety, pharmacokinetic and pharmacodynamic profiles of HP501. Dose proportionality of pharmacokinetic and pharmacodynamic parameters of HP501 was analyzed using a power model by means of a regression analysis with the log-transformed end point as a response and log-dose as a fixed effect. Dose proportionality was supported if the 95% confidence interval (CI) for the slope β fell completely within 0.84–1.16 (the criterion interval: 1 + [ln (0.80)/ln(r)], 1 + [ln (1.25)/ln(r)], r = the highest dose/the lowest dose)^14^. Statistical significance was defined based on a two-tailed *p* value of < 0.05.

### Ethical approval

The study protocol and informed consent forms were reviewed and approved by the Institutional Ethics Committees of the Affiliated Hospital of Southwest Medical University (Luzhou, China; approval number: L2022040) and Chengdu Fifth People’s Hospital (Chengdu, China; approval number: 2021-013-01). The procedures used in this study adhere to the tenets of the Declaration of Helsinki.

### Consent to participate

All participants provided written informed consent before participation in the study.

## Results

### Demographics

**Figure 1:**
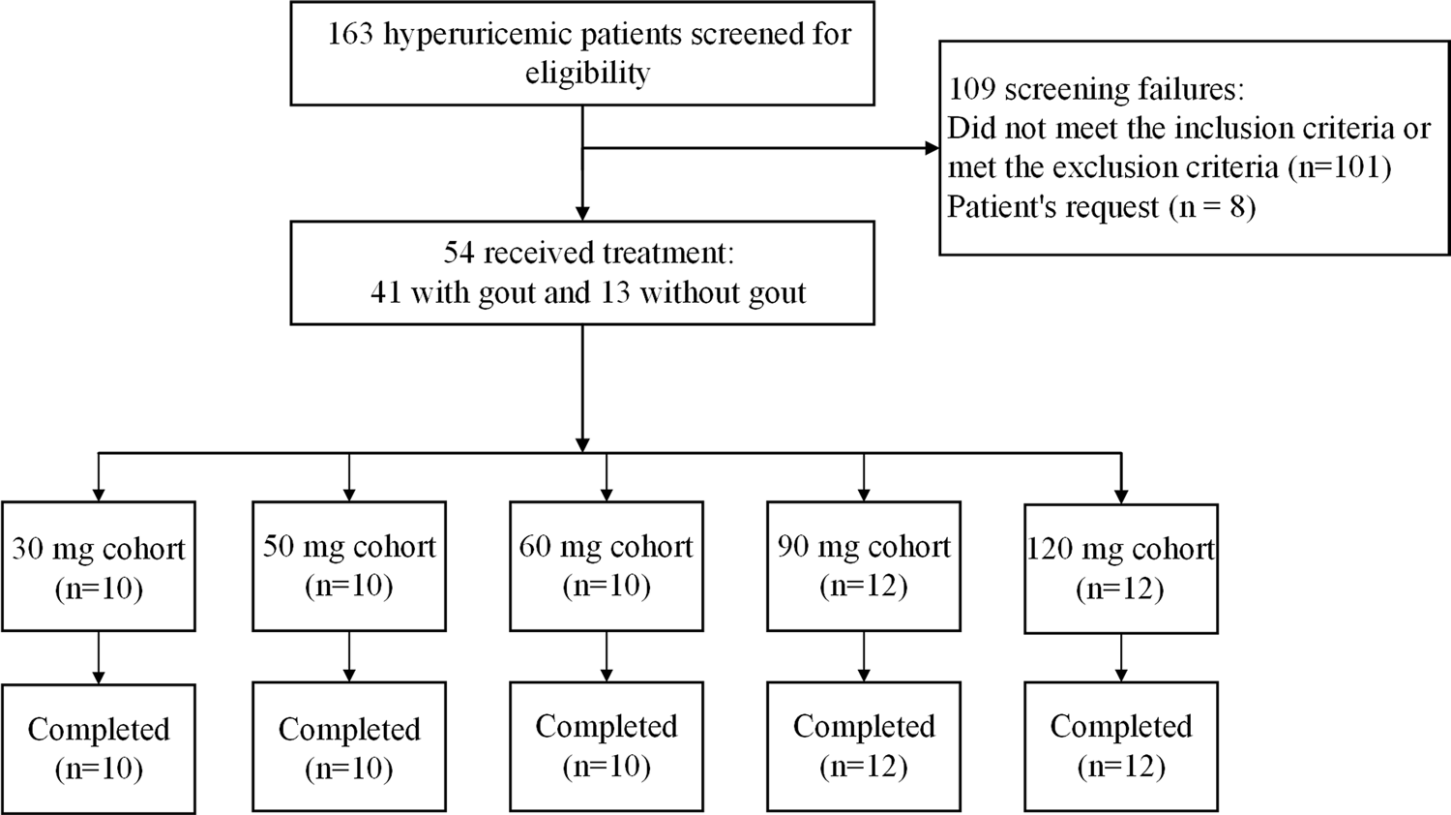
Patient enrollment and treatment assignments.

In the present study, five dose cohorts were used, and 163 patients were screened. Of these 163 patients, 109 were excluded from the study (Fig. 1). Finally, 54 male patients with hyperuricemia aged between 18 and 56 years were included in this study. The 54 patients were sequentially assigned to five dose cohorts (30 mg, 50 mg, 60 mg, 90 mg and 120 mg). All the enrolled patients completed the study. Patient demographics and characteristics are summarized in Table 1.

**Table 1:** Demographic and baseline characteristics of the study patients.

Characteristics	30 mg cohort (n = 10)	50 mg cohort (n = 10)	60 mg cohort (n = 10)	90 mg cohort (n = 12)	120 mg cohort (n = 12)
Age, years					
Mean (SD)	26.3 (5.06)	30.0 (13.22)	29.0 (7.97)	29.6 (6.19)	30.0 (3.72)
Range	20–34	18–56	19–46	22–39	26–37
Sex, n (%)					
Male	10 (100)	10 (100)	10 (100)	12 (100)	12 (100)
Female	0	0	0	0	0
Body weight, kg					
Mean (SD)	71.92 (8.99)	72.93 (9.94)	71.65 (11.68)	73.30 (7.49)	82.38 (7.96)
Range	59.4–89.8	57.4–91.4	52.2–89.2	63.9–83.8	70.2–92.7
Height, cm					
Mean (SD)	169.10 (5.89)	170.25 (4.90)	169.20 (4.90)	170.98 (7.56)	172.71 (5.03)
Range	160.5–179.0	166.0–181.0	163.0–176.0	156.0–184.0	163.0–179.5
BMI, kg/m^2^					
Mean (SD)	25.15 (2.68)	25.10 (2.70)	24.93 (2.70)	25.07 (2.03)	27.58 (2.03)
Range	20.7–28.1	20.8–28.6	20.8–28.6	21.7–29.3	23.8–30.0
Gout history, n (%)	7 (70.00)	9 (90.00)	9 (90.00)	7 (58.33)	9 (75.00)
Serum UA, μmol/L					
Mean (SD)	594.10 (74.42)	672.8 (88.68)	653.2 (100.88)	553.54 (56.31)	616.11 (60.97)
Range	498.0–699.0	549–831	491.0–8155.0	485.9–667.6	538.8–740.4
Classification of hyperuricemia, n (%)					
UA underexcretion	6 (60.00)	5 (50.00)	7 (70.00)	9 (75.00)	12 (100.00)
UA overproduction	0	0	0	0	0
Combined	4 (40.00)	5 (50.00)	3 (30.00)	3 (25.00)	0
Other	0	0	0	0	0

*BMI* Body mass index; *UA* Uric acid; *SD* Standard deviation.

### Safety and tolerability

The AEs reported in this study are summarized in Tables 2 and 3. No death or SAEs were reported in either cohort. No AE leading to the discontinuation of the study was reported. The incidences of AEs in 30 mg, 50 mg, 60 mg, 90 mg and 120 mg cohorts were 60.0% (6/10), 70.0% (7/10), 80.0% (8/10), 75.0% (9/12) and 83.3% (10/12), respectively. The incidences of AEs among the 5 cohorts were not significantly different (*p* = 0.842). The most common AEs (≥ 10% of patients) of any grade regardless of drug relationship were gout flare (14 patients, 25.93%), diarrhea (12 patients, 22.22%), elevated ALT (8 patients, 14.81%), hypertriglyceridemia (7 patients, 12.96%), dry mouth (7 patients, 12.96%) and oral ulcer (7 patients, 12.96%). 14 patients experienced gout flares during the study, which were evaluated as moderate AEs. Other reported AEs in all the 5 cohorts were evaluated as mild. Overall, multiple oral doses of HP501 were well tolerated in all active study cohorts.

**Table 2:** Events of adverse events, n (%).

	30 mg cohort (n = 10)	50 mg cohort (n = 10)	60 mg cohort (n = 10)	90 mg cohort (n = 12)	120 mg cohort (n = 12)
Elevated ALT	1 (10.0)	0	0	3 (25.0)	4 (33.3)
Elevated AST	0	0	0	1 (8.3)	1 (8.3)
Hypertriglyceridemia	0	0	2 (20.0)	1 (8.3)	4 (33.3)
Serum creatinine increased	0	0	0	1 (8.3)	1 (8.3)
Hyperglycemia	0	0	0	1 (8.3)	1 (8.3)
Elevated GGT	1 (10.0)	0	0	0	1 (8.3)
Hypercholesterolemia	0	0	0	0	1 (8.3)
Elevated WBC in blood	0	0	0	0	1 (8.3)
Elevated NEU in blood	0	0	0	0	1 (8.3)
Elevated WBC in urine	1 (10.0)	0	0	0	0
Elevated RBC in urine	0	0	2 (20.0)	0	0
Elevated TBA	0	0	0	0	1 (8.3)
Dry mouth	0	0	0	3 (25.0)	4 (33.3)
Diarrhea	0	3 (30.0)	4 (40.0)	2 (16.7)	3 (25.0)
Oral ulcer	1 (10.0)	2 (20.0)	2 (20.0)	1 (8.3)	1 (8.3)
Gout flare	2 (20.0)	4 (40.0)	5 (50.0)	0	3 (25.0)
Atrial premature beat	0	0	0	1 (8.3)	1 (8.3)
Joint pain	0	0	0	0	1 (8.3)
Epistaxis	0	0	0	0	1 (8.3)
Gingivitis	1 (10.0)	1 (10.0)	0	0	0
Rash	1 (10.0)	1 (10.0)	0	1 (8.3)	1 (8.3)
Dizziness	0	0	1 (10.0)	0	0
Upper respiratory infection	0	0	2 (20.0)	0	0
Hypertension	1 (10.0)	1 (10.0)	2 (20.0)	0	0
Hypotension	1 (10.0)	0	1 (10.0)	0	0
Elevated heart rate	0	0	2 (20.0)	0	0

*ALT* Alanine aminotransferase; *AST* Aspartic transaminase; *GGT* γ-glutamyl transpeptidase; *WBC* White blood cell; *RBC* Red blood cell; *NEU* neutrophile granulocyte; *TBA* Total bile acid.

**Table 3:** Summary of adverse events.

	30 mg cohort (n = 10)		50 mg cohort (n = 10)		60 mg cohort (n = 10)		90 mg cohort (n = 12)		120 mg cohort (n = 12)	
	Number of events, n	Number of patients, n (%)	Number of events, n	Number of patients, n (%)	Number of events, n	Number of patients, n (%)	Number of events, n	Number of patients, n (%)	Number of events, n	Number of patients, n (%)
AE	9	6 (60.0)	16	7 (70.0)	18	8 (80.0)	15	9 (75.0)	36	10 (83.3)
ADR	6	4 (40.0)	9	6 (60.0)	12	6 (60.0)	13	8 (66.7)	29	9 (75.0)
Serious AE	0	0	0	0	0	0	0	0	0	0
Serious ADR	0	0	0	0	0	0	0	0	0	0
AE leading to death	0	0	0	0	0	0	0	0	0	0
AE leading to discontinuation	0	0	0	0	0	0	0	0	0	0

*AE* Adverse event; *ADR* Adverse drug reaction.

### Pharmacokinetic outcomes

**Figure 2:**
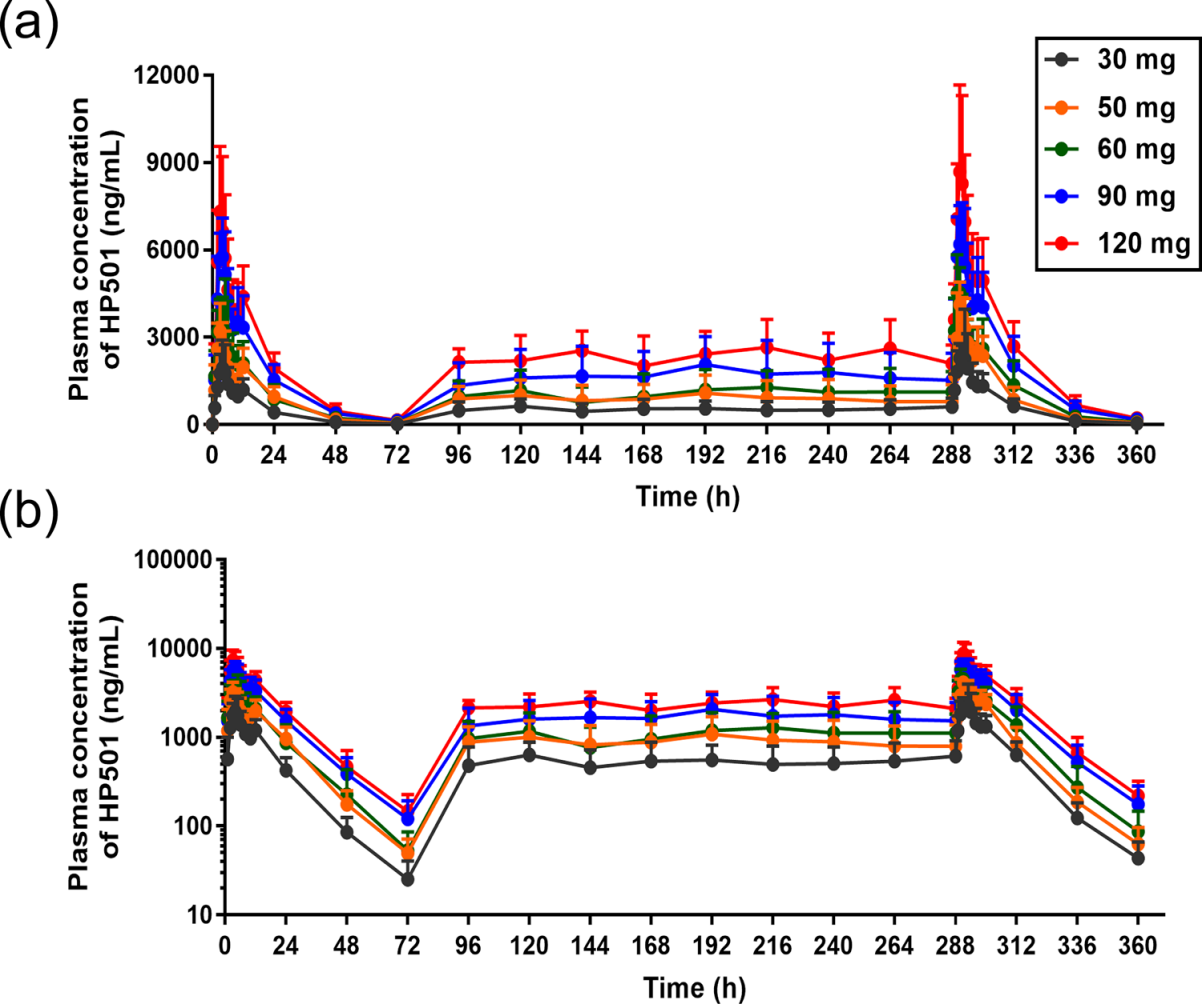
Mean plasma concentrations of HP501 at each time point. (**a**) Linear coordinate; (**b**) semilog coordinate. The error bar indicates the standard deviation.

**Figure 3:**
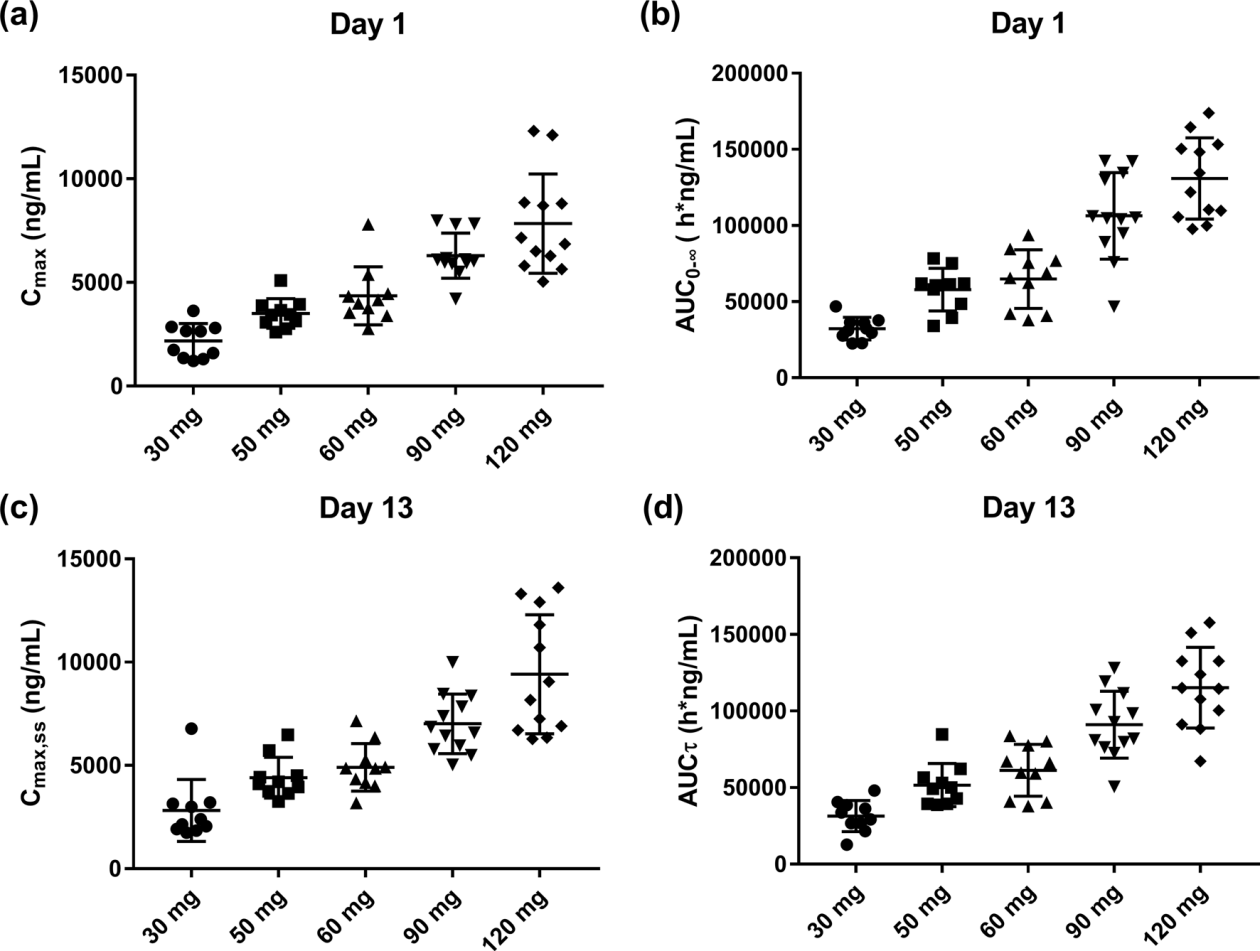
Comparison of pharmacokinetic parameters among five cohorts. (**a**) peak plasma concentration (C_max_); (**b**) area under the concentration-time curve from time zero to infinity (AUC_0-∞_); (**c**) maximal concentration during one dosing interval τ at steady state (C_max,ss_); (**d**) area under the concentration-time curve during one dosing interval τ at steady state (AUC_τ_).

The mean plasma concentration-time curves for HP501 are shown in Fig. 2. Following single dose of 30–120 mg HP501, T_max_ of HP501 occurred at 3.00–4.50 h postdose, suggesting rapid absorption of HP501. The t_1/2_ of HP501 was 10.98–12.53 h. The mean C_max_ in the 30 mg, 50 mg, 60 mg, 90 mg and 120 mg cohorts were 2180.0 ± 836.95 ng/mL, 3504.0 ± 710.54 ng/mL, 4357.0 ± 1396.56 ng/mL, 6294.2 ± 1089.68 ng/mL and 7839.2 ± 2396.65 ng/mL, respectively. The mean AUC_0-∞_ in the 5 cohorts were 32315.713 ± 7371.4244 h*ng/mL, 57970.816 ± 14006.3229 h*ng/mL, 64850.331 ± 19239.5991 h*ng/mL, 106334.098 ± 28391.0542 h*ng/mL and 130866.869 ± 26678.0222 h*ng/mL, respectively. The trends of AUC_0-24 h_ and AUC_0-72 h_ were consistent with those of AUC_0-∞_ (Table 4). In the dose range of 30–120 mg, HP501 exposure increased as the administered dose increased (Fig. 3a-b). Furthermore, the mean CL/F in the 5 cohorts were not significantly different (Table 4).

**Table 4:** Pharmacokinetic parameters of HP501 in single dose period.

Dose	Day	C_max_ (ng/mL)^a^	AUC_0-24_ (h*ng/mL)^a^	AUC_0-72_ (h*ng/mL)^a^	AUC_0-∞_ (h*ng/mL)^a^	T_max_^b^	T_1/2_ (h)^a^	CL/F (mL/h)^a^	Vz/F (mL)^a^
30 mg	1	2180.0 (836.95)	24428.530 (5250.5112)	31872.063 (7082.2738)	32315.713 (7371.4244)	4.50 (2.00–12.00)	11.43 (1.70)	972.71 (222.7993)	15716.775 (2628.4631)
50 mg	1	3504.0 (710.54)	40862.730 (8854.6285)	57170.903 (13719.9440)	57970.816 (14006.3229)	3.00 (3.00–12.00)	10.98 (0.85)	918.034 (263.5715)	14493.479 (4107.0651)
60 mg	1	4357.0 (1396.56)	47442.150 (12260.0054)	63896.025 (18885.7190)	64850.331 (19239.5991)	3.00 (1.00–5.00)	11.58 (2.30)	1015.010 (344.9777)	16803.078 (6043.8993)
90 mg	1	6294.2 (1089.68)	75033.247 (16818.5122)	103991.745 (27106.1987)	106334.098 (28391.0542)	3.49 (2.98–5)	12.53 (1.76)	927.001 (355.2866)	16301.386 (4796.6901)
120 mg	1	7839.2 (2396.65)	91996.904 (19973.3056)	128097.059 (25661.1810)	130866.869 (26678.0222)	3.00 (2.00–5.00)	12.37 (1.53)	952.526 (192.1469)	16818.429 (2966.1415)

*C*_*max*_ Maximum plasma concentration; *AUC*_*0-24 h*_ The area under the plasma concentration-time curves from 0 to 24 h; *AUC*_*0-72 h*_ The area under the plasma concentration-time curves from 0 to 72 h; *AUC*_*0-∞*_ The area under the plasma concentration-time curves from time zero to infinity; *T*_*max*_ Time to peak plasma concentration; T_1/2_, terminal elimination phase half-life; *CL/F* Clearance rate; *Vz/F* Apparent volume of distribution at terminal phase.

^a^expressed as mean (standard deviation).

^b^expressed as median (range).

Following multiple daily doses of 30–120 mg of HP501, a modest increase in HP501 exposure was observed. Plasma concentrations reached steady-state levels after 3 days of dosing. The mean t_1/2_ of HP501 at steady state was 11.77–13.47 h. The mean C_max,ss_ in the 30 mg, 50 mg, 60 mg, 90 mg and 120 mg cohorts were 2819.0 ± 1495.71 ng/mL, 4403.0 ± 985.17 ng/mL, 4911.0 ± 1149.95 ng/mL, 7013.3 ± 1446.60 ng/mL and 9415.8 ± 2883.23 ng/mL, respectively. The mean AUCτ in the 5 cohorts were 31420.175 ± 10183.3767 h*ng/mL, 51638.072 ± 14106.8819 h*ng/mL, 61318.000 ± 16979.2384 h*ng/mL, 91072.444 ± 21854.2916 h*ng/mL, and 115232.837 ± 26369.0376 h*ng/mL, respectively. C_max,ss_ and AUCτ were increased with increasing dose (Fig. 3c,d). The changes of C_min,ss_, AUC_0-72 h_ and AUC_0-∞_ had the similar tendencies (Table 5). Following daily doses of HP501 for 10 days, the mean R_ac_ in the 5 cohorts were 1.306 ± 0.0873, 1.282 ± 0.0426, 1.315 ± 0.1197, 1.413 ± 0.0769 and 1.388 ± 0.0667, respectively. No obvious accumulation of HP501 was observed after daily doses of 30–120 mg of HP501.

**Table 5:** Pharmacokinetic parameters of HP501 in multiple doses period.

Dose	Day	C_max,ss_ (ng/mL)^a^	C_min,ss_ (ng/mL)^a^	AUC_0-72_ (h*ng/mL)^a^	AUC_0-∞_ (h*ng/mL)^a^	AUC_τ_ (h*ng/mL)^a^	Tmax,ss (h)^b^	T_1/2_ (h)^a^	CL/F (mL/h)^a^	Vz/F (mL)^a^	R_ac_^a^
30 mg	13	2819.0 (1495.71)	544.81 (262.20)	42530.531 (13978.2470)	43325.972 (14414.1663)	31420.175 (10183.3767)	3.00 (2.00–5.00)	12.25 (1.71)	1087.544 (500.4241)	19315.080 (10775.4978)	1.306 (0.0873)
50 mg	13	4403.0 (985.17)	718.50 (490.26)	67200.361 (21268.8581)	68301.262 (21825.5280)	51638.072 (14106.8819)	3.00 (2.00–5.00)	12.23 (0.96)	1022.933 (230.2516)	18103.957 (4634.1463)	1.282 (0.0426)
60 mg	13	4911.0 (1149.95)	1037.50 (660.01)	85211.320 (30567.2376)	86719.391 (31575.0910)	61318.000 (16979.2384)	3.00 (1.00–4.00)	11.77 (0.76)	1058.847 (328.9450)	17939.376 (5554.3742)	1.315 (0.1197)
90 mg	13	7013.3 (1446.60)	1417.3 (765.32)	129737.193 (39300.8789)	133266.970 (41550.3425)	91072.444 (21854.2916)	3.00 (1.98–10.00)	13.47 (1.43)	1048.462 (288.5017)	20122.443 (5226.3616)	1.413 (0.0769)
120 mg	13	9415.8 (2883.23)	1972.3 (724.09)	165853.288 (39985.7435)	170114.428 (41905.6715)	115232.837 (26369.0376)	3.00 (2.00–5.00)	13.02 (1.26)	1099.011 (285.8268)	20492.775 (5029.7211)	1.388 (0.0667)

*C*_*max,ss*_ Maximal concentration during one dosing interval τ at steady state; *C*_*min,ss*_ Minimum concentration during one dosing interval τ at steady state; *AUC*_*0-72 h*_ The area under the plasma concentration-time curves from 0 to 72 h; *AUC*_*0-∞*_ The area under the plasma concentration-time curves from time zero to infinity; *AUC*_*τ*_ Area under the concentration-time curve during one dosing interval τ at steady state; *T*_*max*_ Time to peak plasma concentration; *T*_*1/2*_ Terminal elimination phase half-life; *CL/F* Clearance rate; *Vz/F* Apparent volume of distribution at terminal phase; *Rac* Accumulation ratio.

^a^expressed as mean (standard deviation).

^b^expressed as median (range).

The dose-proportionality analysis found that values of β of C_max_ (0.95, 95% CI 0.80–1.11), AUC_0-∞_ (1.02, 95% CI 0.87–1.17), AUC_0-24_ (0.97, 95% CI 0.84–1.10) and AUC_0-72_ (1.01, 95% CI 0.87–1.17) in the SAD part were partially within the criterion interval 0.84–1.16. Similarly, the values of β of C_max,ss_ (0.89, 95% CI 0.73–1.04), C_min,ss_ (1.01, 95% CI 0.67–1.34), AUC_0-∞_ (1.03, 95% CI 0.84–1.22), AUC_0-72_ (1.02, 95% CI 0.83–1.21), and AUCτ (0.96, 95% CI 0.81–1.12) in the MAD part were also partially within the criterion interval.

### Pharmacodynamic outcomes

**Figure 4:**
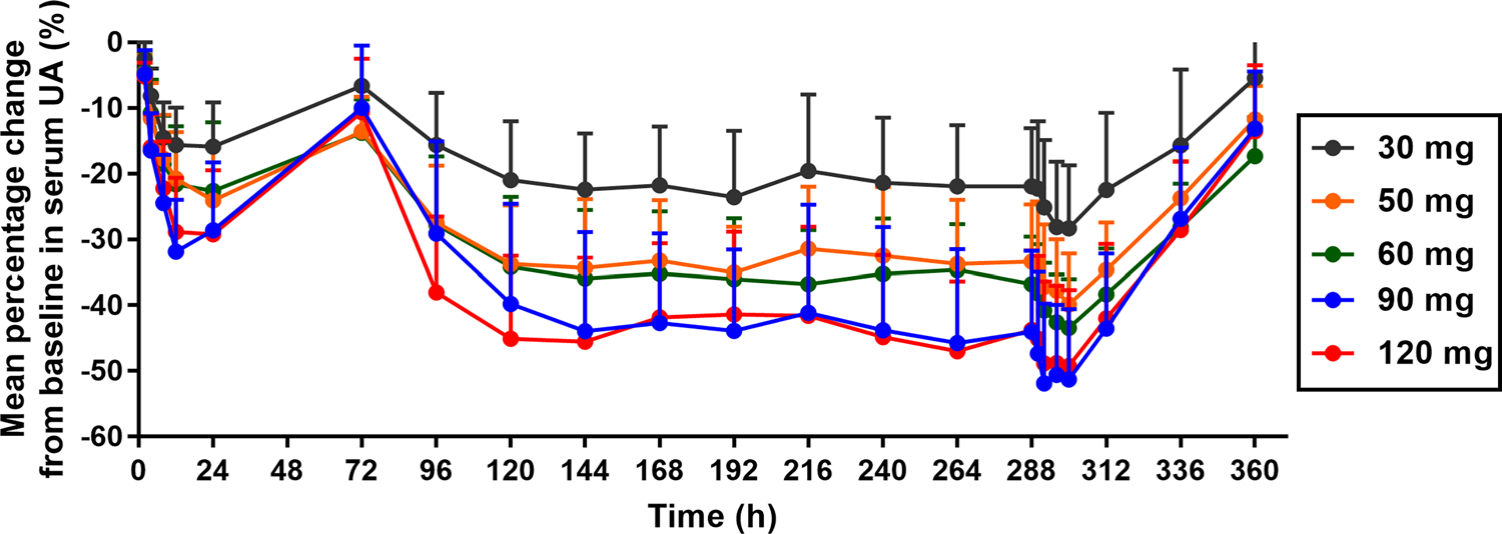
Mean percentage change from baseline in serum UA at each time point. The error bar indicates the standard deviation. UA, uric acid.

The mean percentage changes in serum UA levels from baseline in the five cohorts are shown in Fig. 4. The UA lowering effect of HP501 was increased with an increase in the administration dose from 30 to 90 mg. However, the curve of the 120 mg cohort almost coincided with that of the 90 mg cohort, indicating that the serum UA-lowering effect was similar between the 90 and 120 mg cohorts. Thus, the serum UA lowering effect of HP501 might be saturated at the 90 mg dose level in patients with hyperuricemia.

Following multiple daily doses of 30–120 mg HP501 for 10 days, serum UA levels continued to decline upon repeated dosing. On Day 13, the median T_sUA,max_ was 6.0–8.0 h after dosing. The ΔC_max_% in 30 mg, 50 mg, 60 mg, 90 mg and 120 mg cohorts were 28.6101 ± 9.7988, 40.6595 ± 7.6871%, 43.8074 ± 7.0532%, 52.9132 ± 11.1133% and 50.6123 ± 11.4607%, respectively. ΔAUC_0-24 h_ in the 5 cohorts were 617.783 ± 239.9367 h*%, 890.499 ± 183.2020 h*%, 983.170 ± 169.3917 h*%, 1166.860 ± 266.6979 h*% and 1121.640 ± 279.0327 h*%, respectively. The ΔC_max_% and ΔAUC_0-24 h_ were increased with the increase of the administration dose from 30 to 90 mg. However, the values of the 2 parameters were similar between the 90 mg and 120 mg cohorts. The dose-proportionality analysis found that values of β of ΔC_max_% (0.45, 95% CI 0.29–0.60) and ΔAUC_0-24 h_ (0.48, 95% CI 0.30–0.66) in the MAD part did not fall in the criterion interval 0.84–1.16, indicating HP501 did not possess linear pharmacodynamic characteristics in the dose range of 30–120 mg.

The lowest serum UA level below 360 μmol/L was observed in 2 out of 10 patients (20.00%) in the 30 mg cohort, 5 out of 10 patients (50.00%) in the 50 mg cohort, 5 out of 10 patients (50.00%) in the 60 mg cohort, 10 out of 12 patients (83.33%) in the 90 mg cohort and 9 out of 12 patients (75.00%) in the 120 mg cohort (Table 6). Comparing to 30–60 mg cohorts, 90 mg cohort seemed to have a higher proportion of patients achieving a serum UA level ≤ 360 μmol/L. But the proportion of such patients in the 120 mg cohort was not increased continuously. The 90 mg and 120 mg had similar proportions of patients achieving a serum UA level ≤ 360 μmol/L.

**Table 6:** The number of patients achieving a serum UA level ≤ 360 μmol/L at each time point on Day 13 [n (%)].

Dose	N	Day	0 h	2.0 h	4.0 h	8.0 h	12.0 h	24.0 h	48.0 h	72.0 h
30 mg	10	13	1 (10.00)	1 (10.00)	2 (20.00)	2 (20.00)	2 (20.0)0	1 (10.00)	0	0
50 mg	10	13	3 (30.00)	3 (30.00)	4 (40.00)	3 (30.00)	4 (40.00)	2 (20.00)	0	0
60 mg	10	13	3 (30.00)	3 (30.00)	5 (50.00)	5 (50.00)	5 (50.00)	3 (30.00)	2 (20.00)	0
90 mg	12	13	9 (75.00)	10 (83.33)	10 (83.33)	10 (83.33)	10 (83.33)	10 (83.33)	5 (41.67)	1 (8.33)
120 mg	12	13	8 (66.67)	8 (66.67)	9 (75.00)	8 (66.67)	8 (66.67)	5 (41.67)	2 (16.67)	0

The above results indicate that the serum UA decline was saturated at the 90 mg dose level in patients with hyperuricemia. Owing to the pharmacodynamic outcomes of 30–120 mg cohorts, the UA-lowering effect of HP501 was not expected to increase when the administration dose was over 90 mg. Thus, the 150 mg and 180 mg cohorts were not included in this study.

## Discussion

The present study was conducted based on a former phase I/IIa trial of HP501^12^ with the aim of determining a more suitable administration dose for the phase III trial of HP501. We found that multiple oral doses of 30–120 mg HP501 were well tolerated. Pharmacokinetic analysis showed that HP501 exposure increased with an increase in the administration dose from 30 to 120 mg. However, pharmacodynamic analysis revealed that serum UA lowering might be saturated at the 90 mg dose level in patients with hyperuricemia. The UA-lowering effect of HP501 was not expected to increase when the dose exceeded 90 mg. Thus, the 150 mg and 180 mg cohorts were not studied further.

In the present study, we found that patient with hyperuricemia was well tolerated at a daily dose of 30–120 mg of HP501. No SAEs were reported in either of the cohorts. The most common AEs reported in this study were gout flare, diarrhea, elevated ALT, hypertriglyceridemia, dry mouth and oral ulcer. The incidences of AEs among the 5 active cohorts were not significantly different.

Hepatocellular injury is one of the most common treatment-related AEs associated with uricosuric drugs^15^. During the phase I/IIa trial of HP501, hyperuricemic patients received 45 mg of HP501 for 10 days, and 2/10 and 3/10 patients had elevated AST and ALT levels, respectively^12^. Another phase I trial evaluating the drug-drug interaction of HP501, febuxostat, and colchicine, showed that 1 out of 14 patients who received 40 mg HP501 once daily for 7 days had elevated ALT levels^16^. Due to the previous studies of HP501, we specially focused on the hepatotoxicity of HP501 in this study. We found that only one patient received ≤ 60 mg of HP501 (1/10 in 30 mg cohort) had elevated ALT levels. However, 3/12 patients in the 90 mg cohort and 4/12 in the 120 mg cohort experienced elevated ALT levels. High dose levels of HP501 seemed to have an increasing trend of hepatic injury. Fortunately, similar to the findings of Wang’s study^12^, we found that the liver enzyme activities could return to normal values no more than half a month after the AEs were found without any interruption, indicating the hepatic injury was mild and reversible.

Dry mouth was the most common AE of HP501, as observed in the study conducted by Wang et al.^12^. Our study also observed that of 3/12 patients in the 90 mg cohort and 4/12 in the 120 mg cohort had dry mouth during the study period. AEs associated with dry mouth were mild and temporary. The patient did not require any interruptions.

Nephrotoxicity is regarded as a potential risk factor for URAT1 inhibitors^17,18^ because the increased excretion of UA may lead to its microcrystallization in the renal tubule, causing epithelial injury^7,19^. About 24.30% of patients who received lesinurad (a URAT1 inhibitor developed by Ardea Biosciences) monotherapy had increased serum creatinine^11^. Verinurad, a high-affinity inhibitor of the URAT1 transporter, was reported to elevate serum creatinine 1.5 times to baseline level in 17.1% Japanese patients with hyperuricemia^20^. In the phase I/IIa trial of HP501, no renal-related AEs were observed at study doses ranging from 3 to 60 mg^12^. In the present study, the 90 and 120 mg cohorts showed one case with increased serum creatinine levels, respectively. Of note, the two patients had slightly higher serum creatinine levels at baseline, and the increase in serum creatinine during the study period did not exceed the 1.5 times of normal values. Thus, we could not determine whether the increase in serum creatinine levels in the two cases was related to the study drug. Further studies with longer duration are required to answer this question.

Except for 14 cases of gout flares, all other reported AEs in the five cohorts were mild. Our data showed that 120 mg did not reach the minimum intolerable dose of HP501. The safety of HP501 demonstrated in this study supports the use of 90 or 120 mg of HP501 in subsequent clinical trials.

Our pharmacokinetic results showed that plasma exposure (AUC and C_max_) of HP501 was approximately dose-proportional between 30 and 120 mg in both SAD and MAD. In the phase I/IIa trial, following hyperuricemic patients treated with 45 mg HP501 once daily for 10 days, the C_max_ of serum HP501 was 3.9 ± 1.0 μg/mL and AUC_0-24 h_ was 50.9 ± 10.5 h*μg/mL^12^. Compared to these historical data, hyperuricemic patients receiving 50–120 mg of HP501 for 10 days had a much higher plasma exposure to HP501 in this study. The mean t_1/2_ of HP501 at steady state was 11.77–13.47 h, which is consistent with previous studies^12,16^. Following daily dosing of HP501 for 10 days, the mean R_ac_ was 1.282–1.413 in the 5 cohorts, indicating no obvious accumulation of HP501 during the study period. In the dose-proportionality analysis, the 90% CI of the β values of pharmacokinetic parameters did not completely fall in the criterion interval 0.84–1.16. However, due to the small sample size of this study and the crossover between the 90% CI of β value and the range, it cannot be concluded whether HP501 possesses linear or nonlinear pharmacokinetic characteristics in the dose range of 30–120 mg.

Our pharmacodynamic results did not correspond with the pharmacokinetic results. The curves of serum UA change showed that UA lowering effect of HP501 was increased with an increase in the administration dose from 30 to 90 mg. However, the curve of the 120 mg cohort almost coincided with that of the 90 mg cohort, indicating that the serum UA-lowering effect was similar between the 90 and 120 mg cohorts. The dose-proportionality analysis found that values of β of ΔC_max_% and ΔAUC_0-24 h_ did not fall in the criterion interval 0.84–1.16, indicating HP501 did not possess linear pharmacodynamic characteristics in the dose range of 30–120 mg. The target of serum UA level for the management of hyperuricemia is ≤ 360 μmol/L in consideration of the solubility of urate crystals^21,22^. In this study, 90 mg cohort seemed to have a higher proportion of patients achieving a serum UA level ≤ 360 μmol/L, comparing to 30–60 mg cohorts. However, the percentages of patients achieving a serum UA level ≤ 360 μmol/L were similar between the 90 and 120 mg dose cohorts. These results indicate that the decline of serum UA by HP501 might be saturated at the 90 mg dose level in patients with hyperuricemia. Receptor saturation maybe related to this phenomenon. Based on the pharmacodynamic results, the UA-lowering effect of HP501 was not expected to increase when the administered dose exceeded 90 mg. A once-daily dose of 90 mg HP501 was recommended for subsequent studies. Thus, the 150 mg and 180 mg cohorts were not included in this study.

The present study had several limitations. First, the total treatment duration was short. Further studies are required to evaluate the long-term activity and safety of HP501. Second, it has been reported that the prevalence of hyperuricemia is about 4.9% in Chinese women, which is slightly less prevalent than that in Chinese men (4.9% vs. 7.9%)^23^. This study did not enroll female patients with hyperuricemia. The published literature has shown that the efficacy and safety of treatments for female patients with hyperuricemia cannot be extrapolated from studies that predominantly include males^24^. For example, data from the febuxostat versus Allopurinol Streamlined Trial (FAST) showed that female patients with gout who received XOIs had a higher rate of comorbid cardiovascular risk factors than male patients^25^. Therefore, future studies should evaluate the safety and efficacy of HP501 in female patients. To evaluate the safety and efficacy of HP501 comprehensively, a phase II trial of HP501 is undergoing (CTR20230489), which is scheduled to enroll 120 patients. Finally, the sample size was small and the safety and efficacy of HP501 could not be comprehensively evaluated.

## Conclusion

In summary, multiple oral doses of 30–120 mg HP501 were well tolerated in patients with hyperuricemia with or without gout. Pharmacokinetic analysis showed that HP501 exposure increased with an increase in the administration dose from 30 to 120 mg. However, pharmacodynamic analysis revealed that the serum UA decline was saturated at the 90 mg dose level. Thus, 90 mg HP501 is recommended for subsequent clinical studies of this highly selective URAT1 inhibitor.

## Data availability

The datasets generated during and/or analyzed during the current study are available from the corresponding author on reasonable request.

## Abbreviations

UA: Uric acid
XOIs: Xanthine oxidase inhibitors
URAT1: Urate transporter 1
AEs: Adverse events
SAD: Single ascending dose
MAD: Multiple ascending dose
HEDs: Human equivalent doses
BMI: Body mass index
ALT: Alanine aminotransferase
AST: Aspartate aminotransferase
ULN: Upper limit of normal
ECG: Electrocardiography
SAEs: Serious adverse events
NCA: Non-compartmental analysis
C_max_: Maximum plasma concentration
T_max_: Time to maximum plasma concentration
AUC_0-t_: Area under the concentration-time curve from time zero to the last measurable concentration
AUC_0-∞_: Area under the concentration-time curve from time zero to infinity
t_1/2_: Terminal elimination phase half-life
CL/F: Clearance rate
Vz/F: Apparent volume of distribution at terminal phase
C_max,ss_: Maximal concentration during one dosing interval τ at steady state
C_min,ss_: Minimum concentration during one dosing interval τ at steady state
AUC_τ_: Area under the concentration-time curve during one dosing interval τ at steady state
Rac: Accumulation ratio
